# Identification of potential molecular targets associated with proliferative diabetic retinopathy

**DOI:** 10.1186/s12886-020-01381-5

**Published:** 2020-04-14

**Authors:** Dewang Shao, Shouzhi He, Zi Ye, Xiaoquan Zhu, Wei Sun, Wei Fu, Tianju Ma, Zhaohui Li

**Affiliations:** 1 Department of Ophthalmology, The Chinese People’s Liberation Army General Hospital, No. 28 Fuxing Road, Haidian District, Beijing, 100853 China; 2 Department of Ophthalmology, Air Force Medical Center, PLA, No.15 Chang Yun Gong, Haidian District, Beijing, 100089 China

**Keywords:** Proliferative diabetic retinopathy, Differentially expressed gene, Functional enrichment analysis, microRNA, Angiogenesis

## Abstract

**Background:**

This study aimed to identify and evaluate potential molecular targets associated with the development of proliferative diabetic retinopathy (DR).

**Methods:**

The microarray dataset “GSE60436” generated from fibrovascular membranes (FVMs) associated with proliferative DR was downloaded from the Gene Expression Omnibus database. Differentially expressed genes (DEGs) from the active FVMs and control or inactive FVMs and control were evaluated and co-DEGs were identified using VEEN analysis. Functional enrichment analysis, and protein-protein interactions (PPI) network and module analyses were performed on the upregulated and downregulated coDEGs. Finally, several predictions regarding microRNAs (miRNAs) and transcription factors (TFs) were made to construct a putative TF-miRNA-target network.

**Results:**

A total of 1475 co-DEGs were screened in active/inactive FVM samples, including 461 upregulated and 1014 downregulated genes, which were enriched for angiogenesis [Hypoxia Inducible Factor 1 Subunit Alpha (HIF1A) and Placental Growth Factor (PGF)] and visual perception, respectively. In the case of the upregulated co-DEGs, Kinesin Family Member 11 (KIF11), and BUB1 Mitotic Checkpoint Serine/Threonine Kinase (BUB1) exhibited the highest values in both the PPI network and module analyses, as well as the genes related to mitosis. In the case of downregulated co-DEGs, several G protein subunits, including G Protein Subunit Beta 3 (GNB3), exhibited the highest values in both the PPI network and module analyses. The genes identified in the module analysis were found to be from the signal transduction-related pathways. In addition, we were able to identify four miRNAs and five TFs, including miR-136 and miR-374.

**Conclusions:**

In brief, HIF1A, PGF, KIF11, G protein subunits, and miR-136, miR-374 may all be involved in angiogenesis, retinal endothelial cell proliferation, and visual signal transduction in proliferative DR. This study provides a number of novel insights that may aid the development of future studies dedicated to discovering novel therapeutic targets in proliferative DR.

## Background

Diabetic retinopathy (DR) is one of the most common microvascular complications in diabetes mellitus, and a primary cause of blindness and visual disturbance globally [1]. DR can be divided into non-proliferative and proliferative forms based on the type of microvascular lesions and related ischemic injury [2]. It has been predicted that the global prevalence of diabetes will continue to grow significantly for the next several decades, as a result of increasing incidence of type 1 diabetes rather than increasing type 2 diabetes which has already reached an epidemic level [3]. The incidence of DR increases with diabetic progression and results from a complex process with various molecular and biochemical participants, including oxidative stress, endoplasmic reticulum stress and mitochondrial damage amongst others [4]. The basic mechanisms underlying DR have been extensively studied, however, effective prevention and therapeutic interventions for this disease are still not available.

At present, DR treatments mainly focus on laser photocoagulation, intravitreal pharmacotherapy, anti-VEGF, and glycemic control [5]. Intravitreal anti-inflammatory agents have been effective in treating DR [6], and intravitreal triamcinolone acetonide shows an antiangiogenic effect toward proliferative DR [7]. Vascular endothelial growth factor (VEGF) plays a critical role in promoting vascular permeability, cell migration as well as proliferation of vascular endothelial cells, vasculogenesis, and angiogenesis [8]. VEGF is intimately involved with the progression of proliferative DR and diabetic macular edema facilitating changes in retinal capillary permeability, and advances in anti-VEGF therapies, in age-related macular degeneration, have accelerated the application of anti-VEGF therapies in DR [9]. Oxidative stress is a critical factor in the etiology of DR. Metabolic abnormalities, caused by the increase of glucose concentration in diabetes, can lead to the overproduction of the superoxide radical involved in the uncoupling of mitochondrial electron transport chains, ultimately resulting in oxidative stress [10, 11]. Antioxidants, including Vitamins and polyphenols, are considered a beneficial therapeutic strategy during the treatment of DR. These compounds inhibit the reactive oxygen species, free radicals and enhance the antioxidant defense system [12]. Despite some progress, DR remains a prevalent vision-threatening disease.

In this study, we used the microarray dataset “GSE60436” generated by Ishikawa K et al., [13]. In that study, they identified a subset of differentially expressed genes (DEGs) from active fibrovascular membranes (FVMs) and normal retinas or inactive FVMs and normal retinas, these DEGs were then evaluated by functional analysis. In our study, we aim to identify pivotal genes associated with the pathogenesis of proliferative DR and explore their function and upstream and downstream targets. The common DEGs from active/ inactive FVMs and normal retinas were identified, and then subjected to functional enrichment, protein-protein interaction (PPI) network and module analyses. In addition, the miRNA-target and transcription factors (TFs)-target were predicted to explore the potential regulatory relationships.

## Methods

### Study approval

This study did not use any animal or human participants. All data was generated from public databases.

### Data collection and preprocessing

The gene expression profiling microarray dataset “GSE60436” from FVMs associated with proliferative DR was downloaded from the Gene Expression Omnibus (GEO, http://www.ncbi.nlm.nih.gov/geo/) database. This dataset was made up of a total of nine samples, including three normal human retinas samples (control), three active FVMs samples (age 57.3 ± 2.1 years; male: female, 1:2; duration of type 2 diabetes, 15.7 ± 2.1 years; glycosylated hemoglobin, 8.3%; vitreous hemorrhage, 1; tractional retinal detachment, 2; no anterior chamber neovascularization; no previous anti-VEGF treatment) and three inactive FVM samples (age 48.0 ± 13 years; male: female, 1:2; duration of type 2 diabetes, 12.3 ± 2.5 years; glycosylated hemoglobin, 6.1%; tractional retinal detachment; no anterior chamber neovascularization; no previous anti-VEGF treatment), with sequencing data generated using the GPL6884 Illumina HumanWG-6 v3.0 expression beadchip.

Limma package software (Version 3.10.3, http://www.bioconductor.org/packages/2.9/bioc/html/limma.html) was used to process the raw CEL files downloaded from GEO, and data preprocessing was done using the RMA (robust multi-array average) method including background correction, normalization, and expression calculations. The probes were removed when they were not able to be matched to a specific gene symbol, and the average value was taken as the expression value for each gene when different probes matched to the same gene symbol.

### DEG screening

The genes that were differentially expressed in different groups [active-FVMs vs. control (active group); inactive-FVMs vs. control (inactive group)] were analyzed using the empirical Bayes test from the limma package software [14]. The DEGs were screened using a cut-off value of *P* < 0.05 and |log fold change (FC)| > 2. The DEGs from both groups were then subjected to VEEN analysis using the VENNY (Version 2.1.0, http://bioinfogp.cnb.csic.es/tools/venny/index.html) online tool. Overlapping genes were considered co-regulated DEGs (co-DEGs) in the following analyses.

### Functional enrichment analysis

The Database for Annotation, Visualization and Integrated Discovery (DAVID, Version 6.8, https://david-d.ncifcrf.gov/) online tool was used to analyze the KEGG pathway and Gene Ontology annotations for the co-DEGs. The number of enriched genes was set as: count ≥2, and *P* < 0.05 was considered the threshold value for significantly enriched terms.

### PPI network and module analysis

Search Tool for the Retrieval of Interacting Genes (STRING, Version 11.0, http://www.string-db.org/) was used to predicted the interactions among the co-DEGs with the parameters set to species = human, and PPI score = 0.9 (highest confidence). The PPI network was constructed using Cytoscape software (version 3.2.0, http://www.cytoscape.org/) based on the interactions retrieved from STRING. In addition, the MCODE plugin [15] (Version 1.4.2, http://apps.cytoscape.org/apps/MCODE) from Cytoscape was used to identify the significant modules, with scores > 10, and was followed by functional enrichment analysis.

### TF-miRNA-target network construction

The overrepresentation enrichment analysis method from WebGestalt [16] (http://www.webgestalt.org/) was used to predict the TF- and miRNA-target interactions for all of the genes from the significant modules using a cut-off value of *P* < 0.05. Then, the TF-miRNA-Target regulatory network was constructed by combining miRNA-target and TF-target regulatory interactions.

## Results

### Data preprocessing and DEG screening

**Fig. 1:**
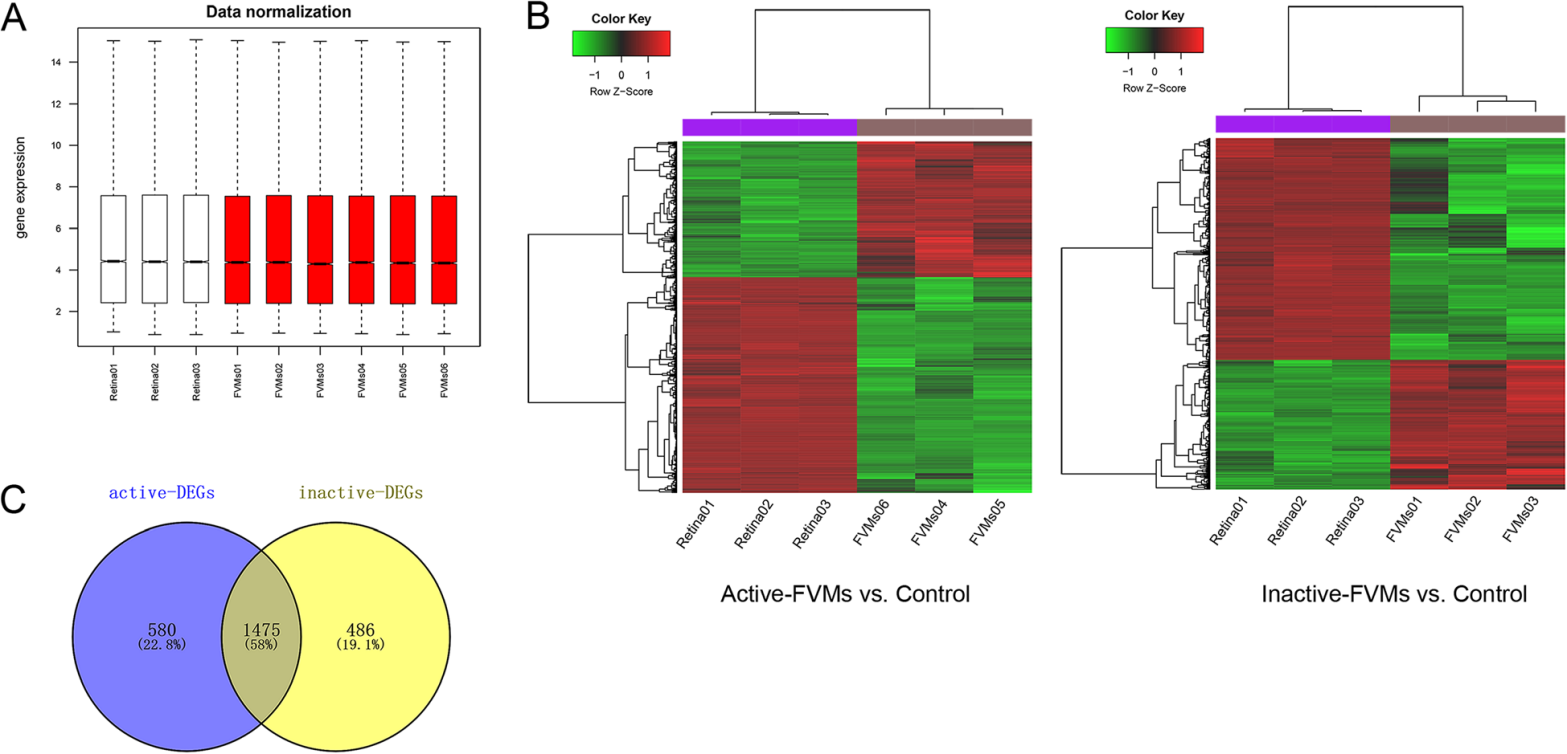
Data normalization and the distribution of differentially expressed genes. **a**, Box plots illustrating data normalization. **b**, Heat maps of DEGs in active and inactive groups. The horizontal axis represents each sample, and the left vertical axis shows clusters of DEGs. The gradual change of color from green to red represents changes in the expression values from low to high. **c**, Venn diagram of DEGs in the two groups. DEGs, differentially expressed genes

The expression values for all the genes from the nine samples were normalized using the RMA method, and values with an unchanged position in the boxplot were used for subsequent analysis, as this can be used as a proxy for normalization (Fig. 1a). A total of 2025 DEGs were identified in the active group, including 758 upregulated DEGs and 1297 downregulated DEGs. Similarly, 1961 DEGs (757 upregulated and 1204 downregulated) were identified in the inactive group. Fig. 1b shows the heat maps of those DEGs, and reveals that the DEGs can be easily distinguished from each of the samples. In addition, the VEEN analysis, which was performed to identify common DEGs in the two groups, identified 1475 overlapping or co-DEGs. Of these 461 genes were upregulated, and 1014 genes were downregulated (Fig. 1c).

### Functional enrichment analysis

The upregulated co-DEGs were significantly enriched for 148 GO-biological processes (GO-BP) and 41 KEGG pathways. Including “GO:0030198~extracellular matrix organization”, “GO:0001525~angiogenesis” and pathways “hsa04512: ECM-receptor interaction”, “hsa04151: PI3K-Akt signaling pathway”. In addition, the downregulated co-DEGs were significantly enriched for 150 GO-BP terms and 40 KEGG pathways including, “GO:0007601~visual perception”, “GO:0001523~retinoid metabolic process” and pathways “hsa04744: Phototransduction” and “hsa04724: Glutamatergic synapse”. Table 1 summarizes the top 10 GO-BP terms and KEGG pathways.

**Table 1:** The top 10 significantly enriched KEGG pathways and GO-BP terms of co-DEGs

(a) Up-regulated co-DEGs			
Category	Terms	*P* value	Genes
GO_BP	GO:0030198~extracellular matrix organization	1.10E-19	PXDN, TNF, LUM, TNC, COL3A1...
GO_BP	GO:0006954~inflammatory response	9.11E-14	C3AR1, CCL3, TNF, NMI, TLR1...
GO_BP	GO:0007155~cell adhesion	4.44E-12	TNC, ITGA11, POSTN, IL32, CXCR3...
GO_BP	GO:0006955~immune response	3.50E-09	PXDN, CCL3, TNF, TLR1, CXCL9...
GO_BP	GO:0001525~angiogenesis	1.37E-08	PIK3CG, COL4A2,PGF, HIF1A, PIK3CA...
GO_BP	GO:0030574~collagen catabolic process	3.59E-08	COL4A2, COL4A1, MMP9, COL3A1, COL6A3...
GO_BP	GO:0030199~collagen fibril organization	4.34E-08	LUM, COL3A1, COL1A2, COL1A1, LOX...
GO_BP	GO:0071347~cellular response to interleukin-1	1.04E-06	ADAMTS7, CCL3, HIF1A, PTGIS, CCL3L1...
GO_BP	GO:0045766~positive regulation of angiogenesis	2.34E-06	C3AR1, CYBB, HIF1A, PTGIS, PGF...
GO_BP	GO:0050900~leukocyte migration	4.37E-06	C3AR1, OLR1, MMP9, F2RL1, ITGA4...
KEGG_pathway	hsa04512:ECM-receptor interaction	6.58E-17	COL4A2, COL4A1, COL3A1, ITGA11, ITGA1...
KEGG_pathway	hsa04510:Focal adhesion	1.43E-12	PGF, TNC, COL3A1, ITGA11, PIK3CA...
KEGG_pathway	hsa04151:PI3K-Akt signaling pathway	3.94E-11	PGF, OSMR, PIK3CA, COL3A1, ITGA11...
KEGG_pathway	hsa04060:Cytokine-cytokine receptor interaction	1.04E-08	CCL3, TNF, TNFSF4, TNFRSF12A, OSMR...
KEGG_pathway	hsa05146:Amoebiasis	5.63E-08	PIK3CG, COL4A2, TNF, COL4A1, COL3A1...
KEGG_pathway	hsa04620:Toll-like receptor signaling pathway	4.16E-07	PIK3CG, IKBKE, CCL3, TNF, CCL3L1...
KEGG_pathway	hsa05222:Small cell lung cancer	1.96E-06	PIK3CG, E2F2, COL4A2, LAMA4, COL4A1...
KEGG_pathway	hsa05162:Measles	5.63E-06	PIK3CG, CD3G, FASLG, CDK6, TLR7...
KEGG_pathway	hsa04640:Hematopoietic cell lineage	1.71E-05	TNF, CD3G, CD36, ITGA5, FCGR1A...
KEGG_pathway	hsa05205:Proteoglycans in cancer	2.71E-05	PIK3CG, TNF, LUM, MMP9, ITGA2...
(b) Down-regulated co-DEGs			
GO_BP	GO:0007601~visual perception	5.92E-40	SLC45A2, PITPNA, MYO7A, RRH, RP1L1...
GO_BP	GO:0001523~retinoid metabolic process	6.62E-11	RBP4, OPN1LW, RBP1, RBP3, OPN1MW...
GO_BP	GO:0006810~transport	7.57E-11	RBP7, CRABP1, GABRB3, GRIK1, RBP1...
GO_BP	GO:0007602~phototransduction	4.43E-10	GUCA1B, UNC119, GUCA1A, TRPC3, OPN1LW...
GO_BP	GO:0042462~eye photoreceptor cell development	7.94E-10	GNAT1, TULP1, CRB1, FSCN2, MYO7A...
GO_BP	GO:0050896~response to stimulus	8.03E-10	SLC45A2, CLDN19, IRX5, SLC24A5, RPGRIP1...
GO_BP	GO:0070588~calcium ion transmembrane transport	4.64E-08	TRPM3, SLC8A2, TRPC3, CACNG7, SLC24A5...
GO_BP	GO:0034220~ion transmembrane transport	1.57E-07	FXYD3, ATP1B1, GRIK1, GABRB3, ATP1B2...
GO_BP	GO:0022400~regulation of rhodopsin mediated signaling pathway	2.34E-07	GUCA1B, GNAT1, PDE6A, GUCA1A, PDE6B...
GO_BP	GO:0042572~retinol metabolic process	3.37E-07	RDH12, RDH8, ALDH1A2, TTR, RBP4...
KEGG_pathway	hsa04744:Phototransduction	3.68E-10	GNAT1, GUCA1B, GUCA1A, GUCA1C, CNGB1...
KEGG_pathway	hsa04724:Glutamatergic synapse	7.66E-08	SLC38A3, GNAO1, GRIK1, ADCY5, GNG13...
KEGG_pathway	hsa04727:GABAergic synapse	1.03E-07	GABRG2, GABARAPL1, SLC38A3, GNAO1, GABRB3...
KEGG_pathway	hsa00350:Tyrosine metabolism	1.48E-07	DCT, DDC, TYRP1, TYR, PNMT...
KEGG_pathway	hsa04723:Retrograde endocannabinoid signaling	1.36E-06	GABRG2, GNAO1, GABRB3, ADCY5, GABRB1...
KEGG_pathway	hsa04721:Synaptic vesicle cycle	1.37E-05	CPLX4, SLC17A7, SLC32A1, ATP6V1C2, SLC17A8...
KEGG_pathway	hsa04261:Adrenergic signaling in cardiomyocytes	2.72E-05	ATP1B1, ATP1B2, MYL3, TNNC1, ADCY5...
KEGG_pathway	hsa05033:Nicotine addiction	4.19E-05	SLC17A7, SLC32A1, SLC17A8, GABRG2, GABRR1...
KEGG_pathway	hsa04020:Calcium signaling pathway	9.36E-05	PTGER1, SLC8A2, SLC25A4, CCKBR, TNNC1...
KEGG_pathway	hsa04725:Cholinergic synapse	3.05E-04	GNAO1, ADCY5, GNG13, KCNJ14, GNG8...

### PPI network and module analysis

For the upregulated co-DEGs.

**Fig. 2:**
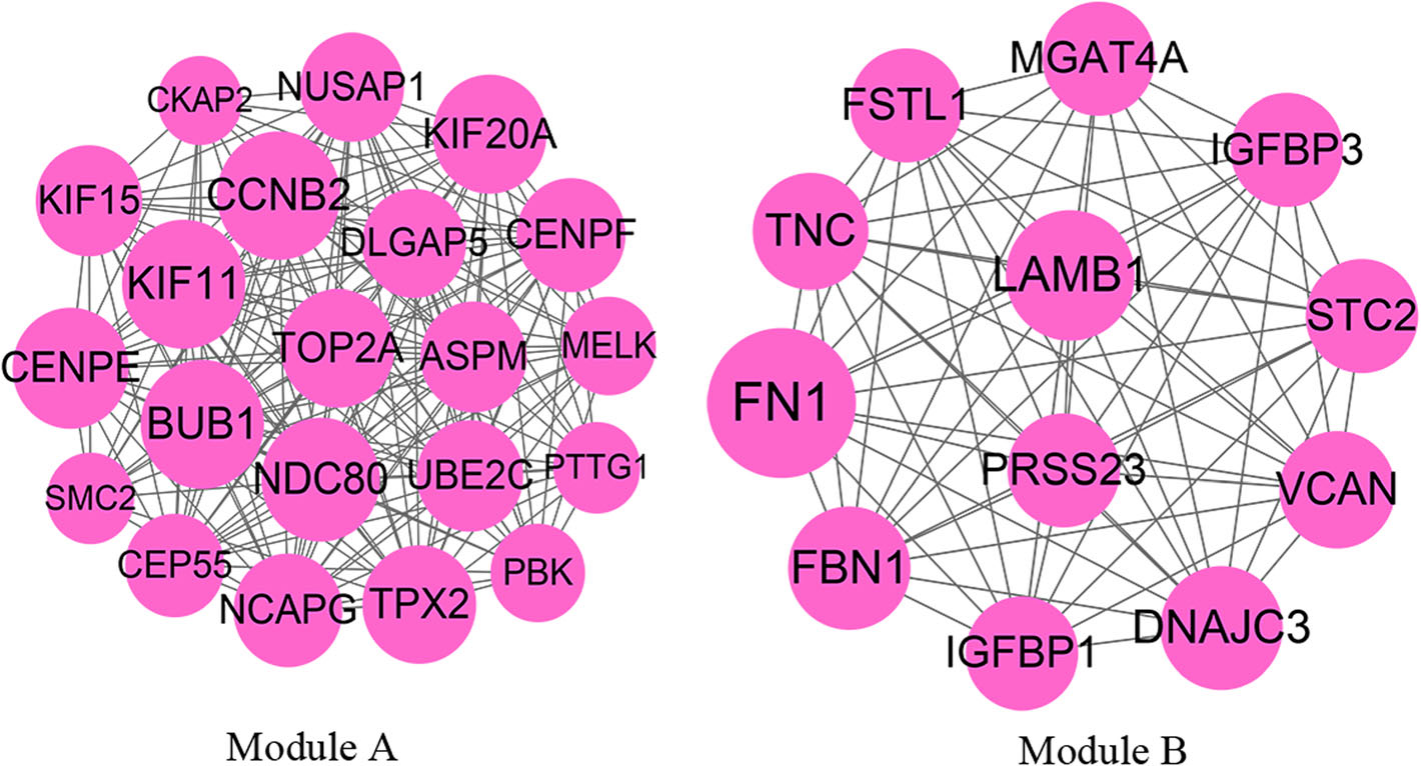
The significant modules identified in the PPI network of upregulated co-DEGs. Node size represents the degree score; lines represent interactions; co-DEGs, co-regulated differentially expressed genes

The upregulated co-DEGs were used to retrieve various predicted interactions, which were then used to create the PPI network. This network consisted of 202 nodes and 751 interactions (Supplemental Fig. 1). Moreover, two significant modules with score ≥ 10 were identified. Module-A (score = 18.4) contained 21 nodes and 184 interactions, and module-B (score = 12) included 12 nodes and 66 interactions (Fig. 2). Kinesin Family Member 11 (KIF11), BUB1 Mitotic Checkpoint Serine/Threonine Kinase (BUB1) and Cyclin B2 (CCNB2) were the hub genes with the highest degree of relevance in both the PPI network and module-A. The genes in module-A were found to be related to three KEGG pathways and 25 GO-BP terms, including “GO:0007067~mitotic nuclear division” and “GO:0051301~cell division”. Additionally, the genes in module-B were found to participated in three KEGG pathways and eight GO-BP terms, including “GO:0030198~extracellular matrix organization”.

For the downregulated co-DEGs.

**Fig. 3:**
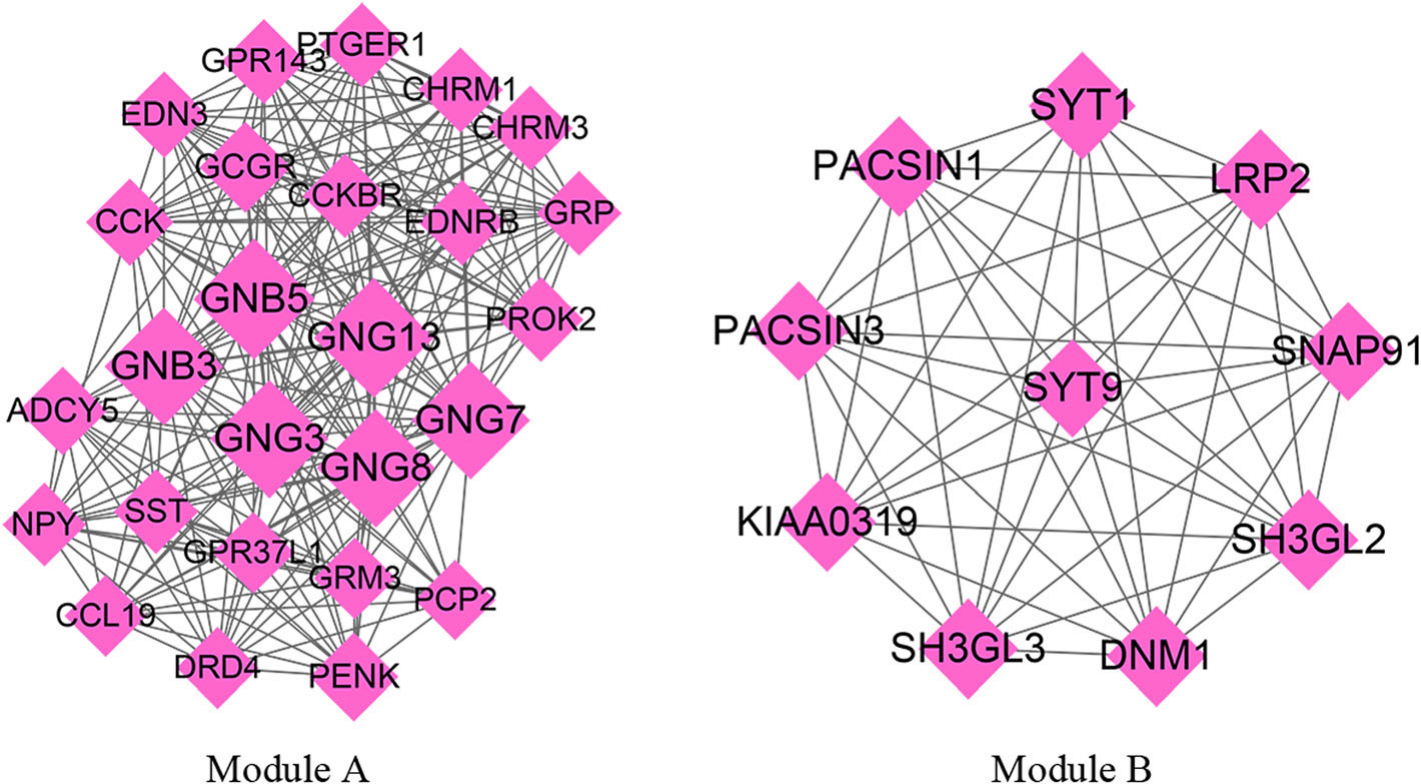
The significant modules identified in the PPI network of downregulated co-DEGs. Node size represents the degree score; lines represent interactions. co-DEGs, co-regulated differentially expressed genes

**Fig. 4:**
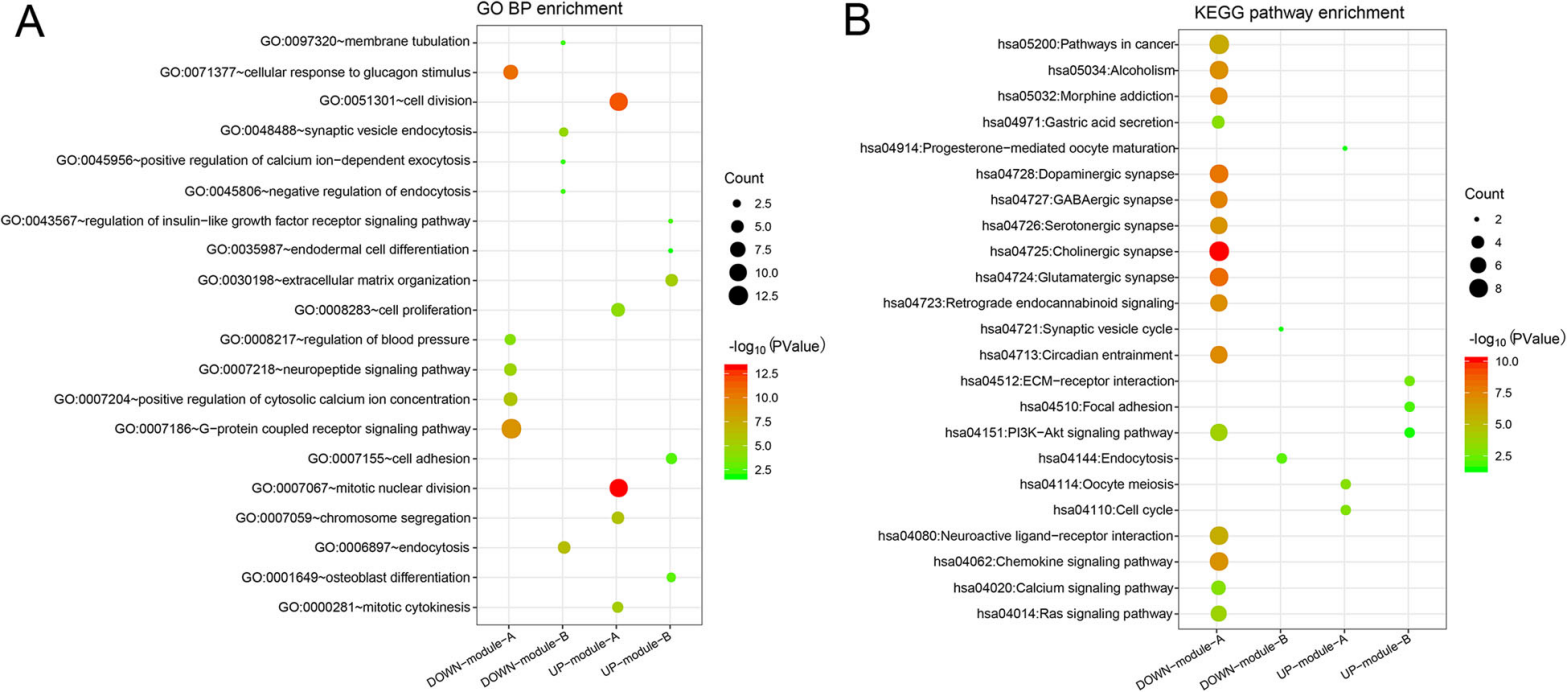
Functional enrichment analysis of the genes found in the significant modules. **a**, top five enriched GO-BP terms **b**, enriched KEGG pathways. The gradual change of color from red to green represents the transition of *p*-values from high to low while bubble size indicates the number of genes enriched in each of the corresponding pathways

The PPI network constructed using downregulated co-DEGs contained 349 nodes and 870 interactions (Supplemental Fig. 2), from which two significant modules with score ≥ 10 were also identified. Module-A (score = 18.24) contained 26 nodes and 228 interactions and module-B (score = 10) included 10 nodes and 45 interactions (Fig. 3). G protein subunits were the hub genes with the highest degree of conservation in both the PPI network and module-A, including G Protein Subunit Beta 3 (GNB3), GNB5, G Protein Subunit Gamma 13 (GNG13), GNG8, GNG17, and GNG3. The genes in module-A were found to be significant in 16 KEGG pathways and 34 GO-BP terms, including “hsa04725: Cholinergic synapse” and “GO:0007186~G−protein coupled receptor signaling pathway”. Similarly, the genes in module-B were found to be associated with two KEGG pathways and nine GO-BP terms. Fig. 4 shows the enriched KEGG pathways and top five GO-BP terms for the genes found in the significant modules.

### TF-miRNA-target network construction

**Fig. 5:**
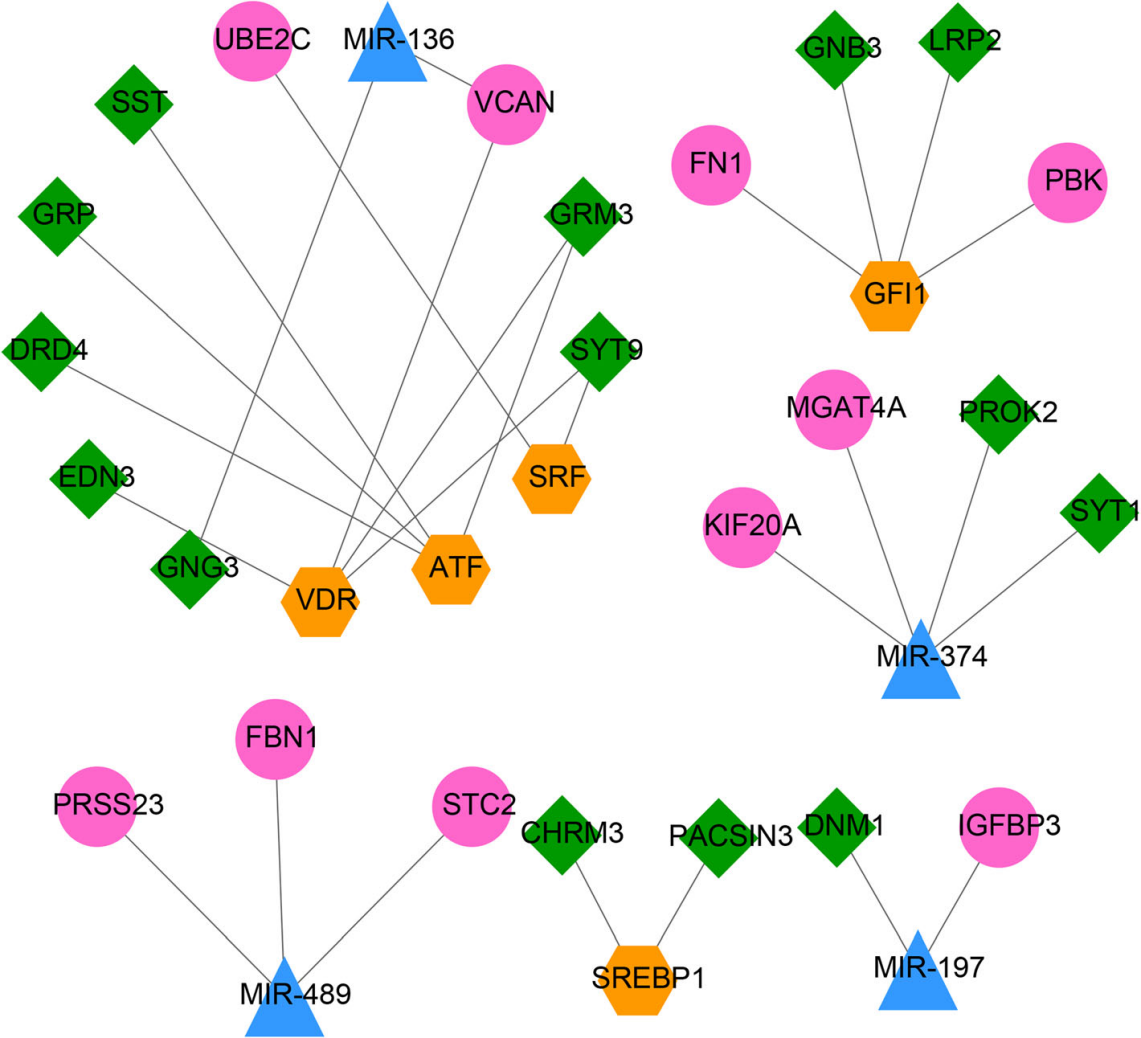
TF-miRNA-Target regulatory network. Blue triangles represent the miRNA; orange hexagons represent TFs; pink circles represent the upregulated genes and green rhombi represent the downregulated genes

The genes from the significant modules were used to predicted the TF- and miRNA-target interactions, which were then used to construct the TF-miRNA-target network (Fig. 5). The TF-miRNA-target network consisted of 33 nodes and 27 interactions, from which we were able to identify four miRNAs and five TFs, which may interact with up to 24 novel genes. GNG3 was shown to be regulated by miR-136, and Growth Factor Independent 1 Transcriptional Repressor (GFI1). KIF20A was regulated by miR-374, and so on.

## Discussion

In this study, we identified 1475 co-DEGs from active/inactive-FVMs samples. The upregulated co-DEGs were found to participate in angiogenesis [Hypoxia Inducible Factor 1 Subunit Alpha (HIF1A) and Placental Growth Factor (PGF)] and mitosis-related processes (the genes in upregulated module-A, including KIF11 and BUB1). The downregulated co-DEGs were enriched in G − protein coupled receptor signaling and other pathways (The genes in downregulated module-A, including GNB3 and GNG8).

The important pathological features of proliferative DR including abnormal growth of retinal blood vessels and angiogenesis have all been linked to VEGF signaling. High blood sugar can trigger hypoxia in retinal tissues, and this hypoxia acts as a crucial factor in the regulation of VEGF-induced angiogenesis through the production of HIF [17]. HIF1A is an hypoxia induced TF and the lack of HIF1A suppresses the formation of the retinal intermediate vascular plexus in mouse models [18]. PGF, a homologue of VEGF, plays an important role in placental development, and has been shown to be involved in pathological angiogenesis both in ocular and non-ocular cancers [19]. In addition, increased expression of PGF has also been observed in the vitreous of DR patients [19]. Bender et al., revealed that PGF and VEGFA are both essential for follicular angiogenesis in primates, and neither can function alone and result in normal follicular angiogenesis [20]. Huang et al. showed that PGF negatively effects retinal endothelial cell barrier function by activating VEGFR1 and VEGFR2 and inhibiting glucose-6-phosphate dehydrogenase and the antioxidant pathways [21]. Ishikawa K et al., reported that the genes that were significantly upregulated in active FVMs were predominantly part of the angiogenesis process [13]. Therefore, we speculate that PGF and VEGF are important in the progression of FVM to proliferative DR.

MicroRNAs (miRNAs) are small non-coding RNAs of around 22 nucleotides (nt) in length, which mediate the expression of protein-coding genes, and are becoming increasingly popular biomarkers used in both prognostic and diagnostic assays [22, 23]. Targets for miR-136 and miR-374 were significantly enriched in the co-DEGs identified in this study. However, the relationship between miR-136 or miR-374 and DR is not well reported, and this is the first publication linking them. miR-136 is highly expressed in pre-eclampsia and inhibits the formation of HUVEC capillaries via dysregulation of VEGF. In addition, the expression levels of miR-136 inversely correlates with VEGF [24]. In paclitaxel-resistant ovarian cancer cells, pre-miR-136 transfection significantly decreased angiogenesis and induced apoptosis, and regulation of miR-136 expression could re-sensitize paclitaxel-resistant cells [25]. C-C Motif Chemokine Ligand 3 represses the expression of miR-374b, which subsequently accelerates VEGF-A level and angiogenesis in osteosarcoma cells [26]. Thus, we speculate that miR-136 and miR-374 may participate in the regulation of angiogenesis in proliferative DR.

KIF11 is a member of the kinesin-like protein family. Mutations in KIF11 have been shown to cause familial exudative vitreoretinopathy associated with retinal detachment [27]. Birtel et al., revealed that KIF11 is important in both ocular development and the maintenance of retinal morphology and function and defects in this protein have been associated with retinal ciliopathy [28]. KIFs are motor proteins involved in the activities of the centrosome and spindle during mitosis. In our study, KIFs (KIF11, KIF15, KIF20A) and other mitosis-associated genes, like BUB1, were significantly enriched in one module involved in cell division and mitotic nuclear division. The proliferation of retinal endothelial cells is a significant event in the progression of DR [29]. In addition, a previous study has shown that high glucose significantly increases the proliferation of retinal endothelial cells and the expression levels of VEGF, also, it was more effective under intermittent high glucose, than constant high glucose conditions [30]. Therefore, we suggest that the genes in this module play a key role in proliferative DR through the regulation of the proliferation of retinal endothelial cells.

G proteins are a kind of heterotrimeric guanine nucleotide-binding protein consisting of three subunits (alpha, beta and gamma subunits), which play an important role in intracellular signal transduction, including insulin, epinephrine, dopamine signaling molecule transduction [31]. GNB3 encodes G Protein Subunit Beta 3. The GNB3 gene polymorphisms, including C825T, have been linked to type 2 diabetes mellitus and its complications [32]. GNB3 knockout lead to the malfunction of cone photoreceptors and ON-bipolar cells in murine retinas [33]. In addition, it was revealed that a naturally occurring mutation in GNB3 results in retinal degeneration in chickens [34]. A homozygous missense variant in GNB3 results in a unique stationary retinal disorder with dual anomalies in visual processing, night blindness and photophobia [35]. G protein subunits (GNB3, GNB5, GNG3, GNG7, GNG8, GNG13) are downregulated in proliferative DR, and significantly enriched in module A which is associated with cellular responses to glucagon stimuli including G − protein coupled receptor signaling pathways and other biological processes. Therefore, we conclude that G proteins may play a crucial role in visual signal transduction in proliferative DR.

Although this study has made several novel observations, all the results were based on bioinformatics analysis, and further experimental verification is required. In addition, in an ideal situation, healthy retinas, non-proliferative diabetic retinas and autologous retinas should be used as controls for a more comprehensive comparison with retinas from subjects with FVM-associated proliferative DR. However, such microarray studies are limited by challenges in obtaining samples. Additionally, the small sample size in this study may also limit the application of our observations. Genes that are important for proliferative DR might be excluded as a result of the small sample size and inter-sample variation.

## Conclusions

In brief, we investigated differentially expressed gene profiles in proliferative DR. Several genes, including HIF1A, PGF, KIF11, G protein subunits, and miR-136, miR-374 were found to be involved in angiogenesis, retinal endothelial cell proliferation, and visual signal transduction in proliferative DR. These findings could provide valuable insight and foundations for the study of proliferative DR’s pathomechanism and may provide an idea of potential therapeutic targets for its treatment.

## Supplementary information

**Additional file 1 Figure S1** The PPI network of upregulated co-DEGs. Node size represents the degree score; lines represent interactions; co-DEGs, co-regulated differentially expressed genes.

**Additional file 2 Figure S2**. The PPI network of downregulated co-DEGs. Node size represents the degree score; lines represent interactions; co-DEGs, co-regulated differentially expressed genes.

## Data Availability

The dataset “GSE60436” were downloaded from GEO data NCBI Gene Expression Omnibus repository. All the data of the current study are available at https://pan.baidu.com/s/12j9WMgvoBapXW3WGCSGfhQ, and the extraction code can be obtained from the corresponding author upon reasonable request.

## Abbreviations

DR: Diabetic retinopathy
FVMs: Fibrovascular membranes
DEGs: Differentially expressed genes
PPI: Protein-protein interactions
miRNAs: Micrornas
TFs: Transcription factors
HIF1A: Hypoxia Inducible factor 1 subunit alpha
PGF: Placental growth factor
KIF11: Kinesin family member 11
BUB1: BUB1 mitotic checkpoint serine/threonine kinase
GNB3: G Protein Subunit Beta 3
VEGF: Vascular endothelial growth factors
co-DEGs: Co-regulated DEGs
GO-BP: GO-biological processes
KIF11: Kinesin family member 11
CCNB2: Cyclin B2
GNG13: G protein subunit gamma 13
GFI1: Growth factor independent 1 transcriptional repressor

## References

[1] Laud K, Shabto U, Tello C: Diabetic Retinopathy. In: Principles of Diabetes Mellitus. edn. Edited by Poretsky L. Cham: Springer International Publishing; 2016:1–18.

[2] Stitt AW, Curtis TM, Chen M, Medina RJ, Mckay GJ, Jenkins A, Gardiner TA, Lyons TJ, Hammes HP, Simó R (2016). The progress in understanding and treatment of diabetic retinopathy. Prog Retin Eye Res.

[3] Duh EJ, Sun JK, Stitt AW (2017). Diabetic retinopathy: current understanding, mechanisms, and treatment strategies. Jci Insight.

[4] Petrovski G, Kaarniranta K, Petrovič D (2017). Oxidative stress, epigenetics, environment, and epidemiology of diabetic retinopathy. Journal of Diabetes Research.

[5] Stewart MW (2016). Treatment of diabetic retinopathy: recent advances and unresolved challenges. World J Diabetes.

[6] Kumar B, Gupta SK, Saxena R, Srivastava S. Current trends in the pharmacotherapy of diabetic retinopathy. J Postgrad Med. 2016;58(2):132–9. 10.4103/0022-3859.97176 22718058

[7] Jonas JB (2007). Intravitreal triamcinolone acetonide for diabetic retinopathy. Dev Ophthalmol.

[8] Gupta N, Mansoor S, Sharma A, Sapkal A, Sheth J, Falatoonzadeh P, Kuppermann B, Kenney M (2013). Diabetic retinopathy and VEGF. Open Ophthalmol J.

[9] Nicholson BP, Schachat AP (2010). A review of clinical trials of anti-VEGF agents for diabetic retinopathy. Graefes Arch Clin Exp Ophthalmol.

[10] Calderon GD, Juarez OH, Hernandez GE, Punzo SM, ZDDL C. Oxidative stress and diabetic retinopathy: development and treatment. Eye. 2017;31(8):1122–30. 10.1038/eye.2017.64 PMC5558229 28452994

[11] Guzman DC, Olguín HJ, García EH, Peraza AV, Dz DLC, Soto MP (2017). Mechanisms involved in the development of diabetic retinopathy induced by oxidative stress. Redox Rep.

[12] Li C, Miao X, Li F, Wang S, Liu Q, Wang Y, Sun J. Oxidative Stress-Related Mechanisms and Antioxidant Therapy in Diabetic Retinopathy. Oxid Med Cell Longev. 2017;(11):9702820. 10.1155/2017/9702820 PMC5317113 28265339

[13] Keijiro I, Shigeo Y, Yoshiyuki K, Yedi Z, Takahito N, Shintaro N, Yukio S, Yuji O, Hiroaki N, Koichi A (2015). Microarray analysis of gene expression in fibrovascular membranes excised from patients with proliferative diabetic retinopathy. Invest Ophthalmol Vis Sci.

[14] Smyth G.K. (2005) limma: Linear Models for Microarray Data. In: Gentleman R, Carey VJ, Huber W, Irizarry RA, Dudoit S. (eds) Bioinformatics and Computational Biology Solutions Using R and Bioconductor. Statistics for Biology and Health. Springer: New York.

[15] Bandettini WP, Kellman P, Mancini C, Booker OJ, Vasu S, Leung SW, Wilson JR, Shanbhag SM, Chen MY, Arai AE (2012). MultiContrast delayed enhancement (MCODE) improves detection of subendocardial myocardial infarction by late gadolinium enhancement cardiovascular magnetic resonance: a clinical validation study. J Cardiovasc Magn Reson Official J Soc Cardiovasc Magn Reson.

[16] Nucleic Acids Res. 2005, 33(Web Server issue):W741–8. 10.1093/nar/gki475 PMC1160236 15980575

[17] Crawford TN, Rd AD, Kerrison JB, Jablon EP. Diabetic retinopathy and angiogenesis. Curr Diabetes Rev. 2009;5(1):8–13. 10.2174/157339909787314149 19199892

[18] Christian C, Markus T, Christina L, Sandrine J, Marijana S, Christian G (2011). HIF1A is essential for the development of the intermediate plexus of the retinal vasculature. Invest Ophthalmol Vis Sci.

[19] Nguyen QD, De FS, Behar-Cohen F, Lam WC, Li X, Reichhart N, Ricci F, Pluim J, Li WW (2018). Placental growth factor and its potential role in diabetic retinopathy and other ocular neovascular diseases. Acta Ophthalmol.

[20] Bender HR, Trau HA, Duffy DM (2018). Placental growth factor is required for ovulation, Luteinization, and angiogenesis in primate ovulatory follicles. Endocrinology.

[21] Huang H, Lennikov A, Saddala MS, Gozal D, Grab DJ, Khalyfa A, Fan L (2019). Placental growth factor negatively regulates retinal endothelial cell barrier function through suppression of glucose-6-phosphate dehydrogenase and antioxidant defense systems. FASEB J.

[22] Joglekar MV, Januszewski AS, Jenkins AJ, Hardikar AA (2016). Circulating microRNA biomarkers of diabetic retinopathy. Diabetes.

[23] Farr RJ, Januszewski AS, Joglekar MV, Liang H, McAulley AK, Hewitt AW, Thomas HE, Loudovaris T, Kay TWH, Jenkins A (2015). A comparative analysis of high-throughput platforms for validation of a circulating microRNA signature in diabetic retinopathy. Sci Rep.

[24] Ji L, Zhang L, Li Y, Guo L, Cao N, Bai Z, Song Y, Xu Z, Zhang J, Liu C (2017). MiR-136 contributes to pre-eclampsia through its effects on apoptosis and angiogenesis of mesenchymal stem cells. Placenta.

[25] An H, Jeong J, Lee M, Song J, Jung Y, Kim Y, Kwon A, Huh J, Kim K, Kang H (2014). 275: MiR-136 targeting Notch3 is involved in chemoresistance and angiogenesis in ovarian cancer cells. Eur J Cancer.

[26] Liao Y-Y, Tsai H-C, Chou P-Y, Wang S-W, Chen H-T, Lin Y-M, Chiang IP, Chang T-M, Hsu S-K, Chou M-C (2015). CCL3 promotes angiogenesis by dysregulation of miR-374b/ VEGF-A axis in human osteosarcoma cells. Oncotarget.

[27] Hu H, Xiao X, Li S, Jia X, Guo X, Zhang Q (2016). KIF11 mutations are a common cause of autosomal dominant familial exudative vitreoretinopathy. Br J Ophthalmol.

[28] Birtel J, Gliem M, Mangold E, Tebbe L, Spier I, Müller PL, Holz FG, Neuhaus C, Wolfrum U, Bolz HJ (2017). Novel insights into the Phenotypical Spectrum of KIF11-associated retinopathy, including a new form of retinal Ciliopathy. Invest Ophthalmol Vis Sci.

[29] West DC, Kumar S (1988). Endothelial cell proliferation and diabetic retinopathy. Lancet.

[30] Sun J, Xu Y, Sun S, Sun Y, Wang X (2010). Intermittent high glucose enhances cell proliferation and VEGF expression in retinal endothelial cells: the role of mitochondrial reactive oxygen species. Mol Cell Biochem.

[31] Gilman AG (1986). G proteins: transducers of receptor-generated signals. Annu Rev Biochem.

[32] Rizvi S, Raza ST, Rahman Q, Mahdi F (2016). Role of GNB3 , NET , KCNJ11 , TCF7L2 and GRL genes single nucleotide polymorphism in the risk prediction of type 2 diabetes mellitus. Biotech.

[33] Arno G, Holder GE, Chakarova C, Kohl S, Pontikos N, Fiorentino A, Plagnol V, Cheetham ME, Hardcastle AJ, Webster AR (2016). Recessive retinopathy consequent on mutant G-protein β subunit 3 (GNB3). Jama Ophthalmol.

[34] Hemanth T, Manir A, Paul G, Hocking PM, Burt DW, Inglehearn CF, Lester DH (2006). Mutation in the guanine nucleotide-binding protein beta-3 causes retinal degeneration and embryonic mortality in chickens. Invest Ophthalmol Vis Sci.

[35] Banin E, Bocquet B, Debaere E, Casteels I, Defoort-Dhellemmes S, Drumare I, Friedburg C, Gottlob I, Jacobson S, Kellner U (2016). Biallelic mutations in GNB3 cause a unique form of autosomal-recessive congenital stationary night blindness. Am J Hum Genet.

