# Supplementary figures and images for: Identification of potential molecular targets associated with proliferative diabetic retinopathy
Source: BMC Ophthalmol. 2020 Apr 14;20:143. doi: 10.1186/s12886-020-01381-5 (PMC7155274; doi:10.1186/s12886-020-01381-5)

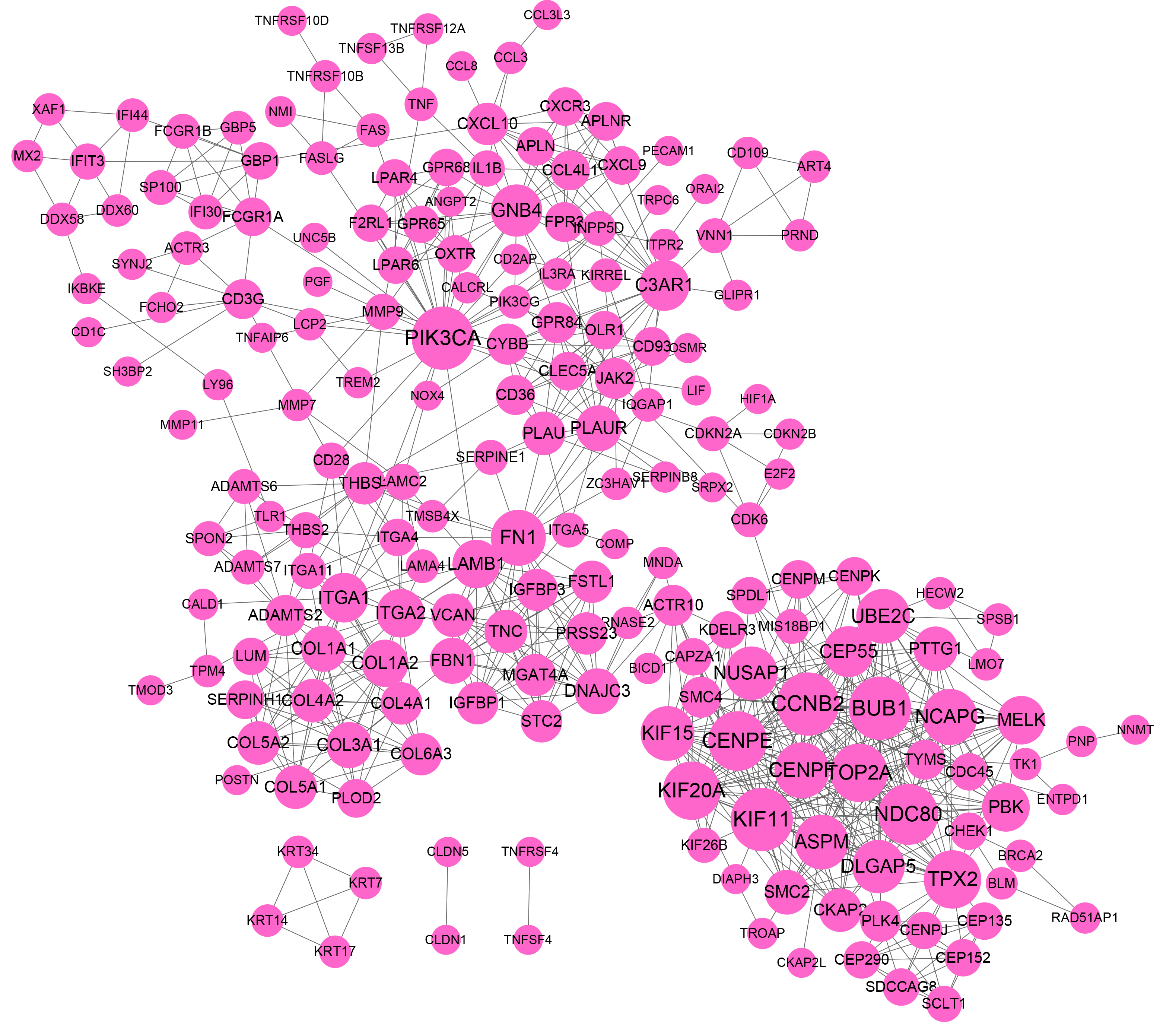

Supplement: Supplementary file 1 — Additional file 1 Figure S1 The PPI network of upregulated co-DEGs. Node size represents the degree score; lines represent interactions; co-DEGs, co-regulated differentially expressed genes. [file 12886_2020_1381_MOESM1_ESM.tif]

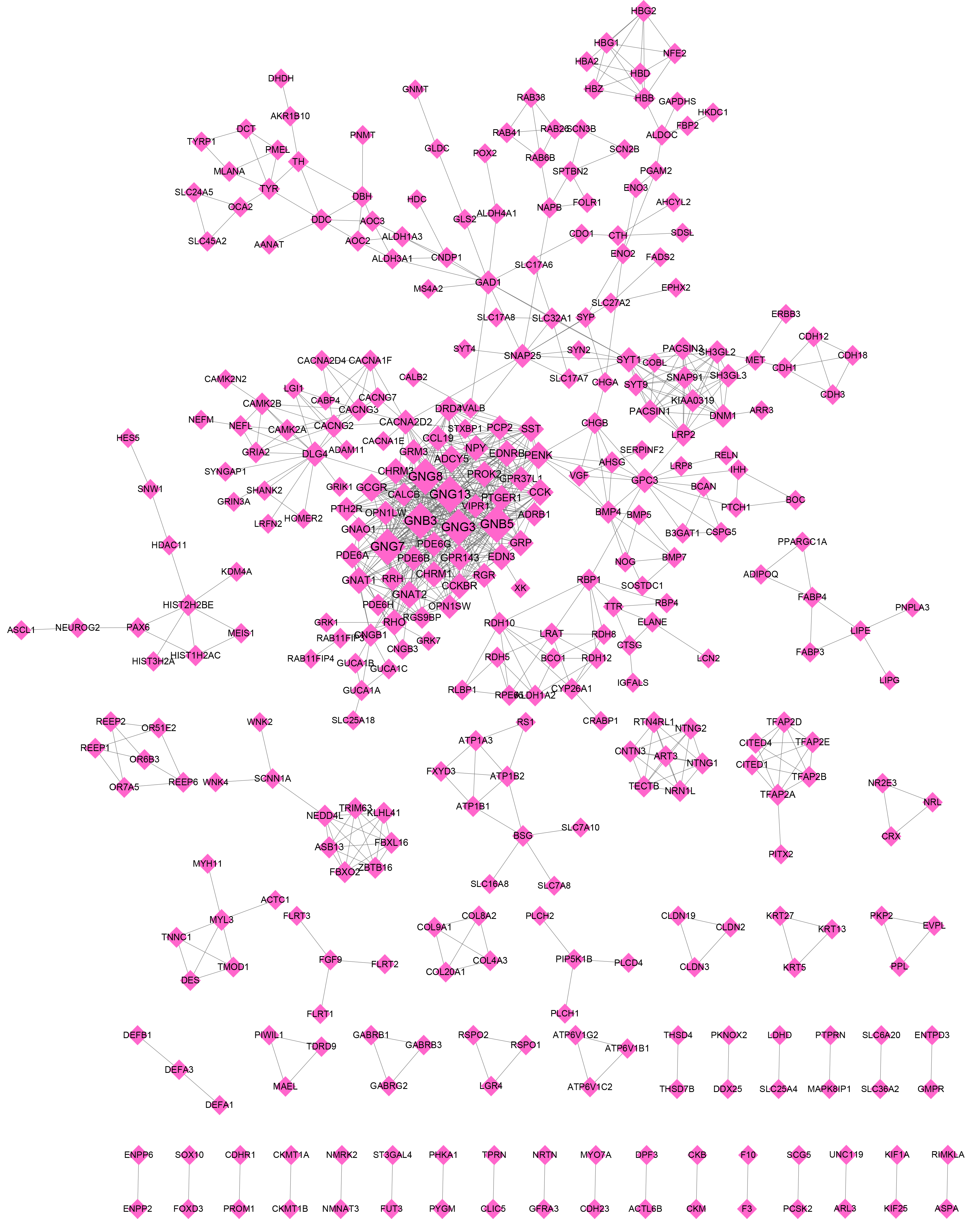

Supplement: Supplementary file 2 — Additional file 2 Figure S2. The PPI network of downregulated co-DEGs. Node size represents the degree score; lines represent interactions; co-DEGs, co-regulated differentially expressed genes. [file 12886_2020_1381_MOESM2_ESM.tif]
